# The CODYSUN Approach: A Novel Distributed Paradigm for Dynamic Spectrum Sharing in Satellite Communications

**DOI:** 10.3390/s21238052

**Published:** 2021-12-02

**Authors:** Irfan Jabandžić, Fadhil Firyaguna, Spilios Giannoulis, Adnan Shahid, Atri Mukhopadhyay, Marco Ruffini, Ingrid Moerman

**Affiliations:** 1 IDLab, Department of Information Technology, Ghent University–imec, 9052 Ghent, Belgium; irfan.jabandzic@ugent.be (I.J.); spilios.giannoulis@ugent.be (S.G.); adnan.shahid@ugent.be (A.S.); 2 CONNECT Centre, Trinity College Dublin, 2 Dublin, Ireland; firyaguf@tcd.ie (F.F.); amukhopa@tcd.ie (A.M.); marco.ruffini@tcd.ie (M.R.)

**Keywords:** satellite communications, dynamic spectrum sharing, spectrum coexistence, distributed algorithms, ns-3

## Abstract

With a constant increase in the number of deployed satellites, it is expected that the current fixed spectrum allocation in satellite communications (SATCOM) will migrate towards more dynamic and flexible spectrum sharing rules. This migration is accelerated due to the introduction of new terrestrial services in bands used by satellite services. Therefore, it is important to design dynamic spectrum sharing (DSS) solutions that can maximize spectrum utilization and support coexistence between a high number of satellite and terrestrial networks operating in the same spectrum bands. Several DSS solutions for SATCOM exist, however, they are mainly centralized solutions and might lead to scalability issues with increasing satellite density. This paper describes two distributed DSS techniques for efficient spectrum sharing across multiple satellite systems (geostationary and non-geostationary satellites with earth stations in motion) and terrestrial networks, with a focus on increasing spectrum utilization and minimizing the impact of interference between satellite and terrestrial segments. Two relevant SATCOM use cases have been selected for dynamic spectrum sharing: the opportunistic sharing of satellite and terrestrial systems in (i) downlink Ka-band and (ii) uplink Ka-band. For the two selected use cases, the performance of proposed DSS techniques has been analyzed and compared to static spectrum allocation. Notable performance gains have been obtained.

## 1. Introduction

In the present day, the spectrum usage of satellite communications (SATCOM) is tightly regulated (mainly based on planned spatial and frequency separation), leading to static and fixed spectrum allocation. Such an approach works well, as long as the number of satellite operators and satellites is limited, and the bandwidth requirements of the services are not too high. However, the number of satellites that will be launched in the next few years is expected to grow rapidly, from projects such as Starlink, oneWeb, etc. For instance, SpaceX as of 24 October 2020 has launched 895 Starlink satellites. Furthermore, they plan to launch nearly 12,000 satellites with a possible extension to 42,000 satellites [1]. Considering the increasing satellite density, the static and fixed spectrum allocation model is not sustainable and will lead to a shortage of spectrum. Fixed spectrum assignment will eventually yield poor performance either due to interference or poor spectrum utilization.

Equivalent power flux density (EPFD) rules [2] for interference management in SATCOM are still working fine for low-density scenarios. However, these rules are only designed for protecting static geostationary (GSO) satellites from interference generated by non-geostationary (NGSO) satellites or other GSO satellites. In addition, these rules are very conservative as they apply a big protection margin that seriously limits the accommodation of new NGSO satellites. To overcome this, it becomes imperative to reuse and share the spectrum by moving towards spectrum sensing and dynamic spectrum sharing (DSS) for improving spectrum utilization and mitigating the impact of interference in high-density SATCOM scenarios.

In SATCOM, traditionally, fixed satellite services (FSS) use C and K bands whereas mobile satellites use L and S bands. Due to the limited availability of L and S bands and the continuously increasing demands of broadband services via mobile satellites, Ku and Ka bands have also been assigned to mobile satellite services. Interference management is a challenging task in satellite systems in which two kinds of satellites, i.e., GSO and NGSO operate in overlapping coverage areas in the same frequency bands [3]. This situation becomes much more complicated due to many systems with thousands of such satellites all sharing the same frequency bands. Additionally, the complexity of interference management is significantly increased if satellites communicate with mobile earth stations in motion (ESIMs).

There has been tremendous pressure on satellite bands due to the launch of new terrestrial services including mobile telephony, WiMAX, 5G, etc. Uplink Ka-band is one of the main target bands for the mmWave (FR2) operation of the 5G New Radio (NR) specification. Both Korea in June 2018 and United States in January 2019 concluded spectrum auctions on uplink Ka-band for terrestrial 5G services [4]. This introduction of terrestrial services in bands used by satellite services further emphasizes the need for migration from fixed to dynamic spectrum assignment and demands coexistence not only between multiple satellites but also with terrestrial services, when operating in the same bands.

There are several SATCOM scenarios that may benefit from DSS. Recently, Ka-band has been gaining interest because of the more mature and cost-effective technology, and because this part of the spectrum is still less congested than the more traditional C and Ku bands [4]. However, it is anticipated that limited resources, expanding Internet use, and increasing density of satellite deployments will complicate future Ka-band deployments [5]. The European Conference of Postal and Telecommunications Administrations (CEPT) has adopted a decision for downlink communication in Ka-band (17.7–19.7 GHz), ERC/DEC/(00)07 [6], which gives guidance on the use of this band by FSS and terrestrial networks. According to this decision, FSS stations can be deployed anywhere but without the right of protection from interference generated by fixed service (FS) terrestrial links. DSS techniques could significantly increase the FSS spectrum usage by utilizing spatial, time, and/or frequency separation with dynamic (re)allocation of spectrum resources to protect FSS from interference by other FSS and terrestrial links. By CEPT decision ECC/DEC/(05)01 [7], a segmentation is provided between FSS and terrestrial stations in uplink Ka-band (27.5–29.5 GHz). As the terrestrial segment of this band is underutilized throughout Europe, FSS stations could maximize their spectrum utilization by dynamically exploiting the spectrum allocated to FSS and terrestrial segments of the uplink Ka-band. At the same time, the DSS techniques need to provide interference protection of terrestrial incumbent users, as well as mutual interference protection of FSS links.

European Space Agency (ESA) has recognized that the current fixed spectrum sharing model is not a feasible approach for the future of SATCOM and, as a potential replacement, they have pinpointed a dynamic sharing of the spectrum. ESA made clear that this dynamic spectrum sharing research endeavor should take a clean slate approach, breaking free from the constraints that existing SATCOM regulations impose. It is aiming to be the driving force behind the adaptation of existing regulations towards a new era of dynamic spectrum management, and not a mere upgrade on the current regulatory framework that is aimed mainly towards static spectrum assignments and centralized control. To reach that goal, the benefits of applying DSS techniques in SATCOM need to be examined first and compared to the existing spectrum sharing model. If the achieved gains are sufficient, the migration from fixed to dynamic spectrum sharing can be considered, and existing regulations adapted accordingly. In order to increase spectrum utilization while still guaranteeing interference-free operation, we investigated the effects of novel DSS techniques in the Ka-band SATCOM in terms of performance gains and mitigation of interference components. Being mandated by ESA, we carried out the CODYSUN (COllaborative and DYnamic approaches to increasing Spectrum UtilizatioN) [8] study in order to investigate the current status quo of SATCOM and propose ways to enable advanced DSS techniques for solving the spectrum crunch problems in SATCOM. Therefore, in CODYSUN, we identified shortcomings of existing SATCOM solutions and proposed novel DSS techniques to avoid interference and reach the highest possible level of spectrum efficiency and reuse by exploiting all possible degrees of freedom (frequency, time, and space). This paper discloses for the first time the proposed techniques and achieved results from the CODYSUN study.

The DSS techniques investigated in the CODYSUN study have been inspired during our participation in the Defense Advanced Research Projects Agency (DARPA) Spectrum Collaboration Challenge (SC2) [9], where, as part of team SCATTER [10], we designed and implemented DSS techniques for terrestrial networks. The goal of SC2 was to ensure that the exponentially growing number of military and civilian wireless devices and services would have full access to the increasingly crowded electromagnetic spectrum. By breaking the walls between isolated silos of the exclusively assigned spectrum, and offering a wider shared spectrum band to multiple independent and collaborative networks, current problems of underutilization of spectrum in exclusive spectrum bands, leading to a huge waste of spectrum, can be mitigated. By exploiting the spatial separation, the same spectrum can be simultaneously used by multiple communication entities, heavily boosting spectrum utilization efficiency. Starting from the results obtained in DARPA SC2 for terrestrial networks, the CODYSUN study has revealed that similar DSS techniques can be applied for spectrum sharing across both satellite and terrestrial deployments.

The main contributions of this paper can be summarized as follows:

1. From several initially assessed use cases, after careful analysis and consultation with ESA, we selected the two most prominent use cases to be analysed. The list of selected use-cases was subsequently also approved by ESA, which was mandatory in order to move forward. The selected use cases were: (1) opportunistic sharing of GSO/NGSO satellite and terrestrial systems in downlink Ka-band; and (2) opportunistic sharing of GSO/NGSO satellite and terrestrial systems in uplink Ka-band. The major criterion for selecting relevant dynamic use cases was the presence of three systems (GSO, NGSO, and terrestrial networks) operating in the same frequency band. In comparison, current research on applying DSS techniques in SATCOM is focused on use cases where only two systems share the spectrum.
2. To solve the interference and spectrum underutilization problems in selected use cases, two DSS techniques were presented: (1) DSS1—collaboration protocol; and (2) DSS2—decentralized spectrum sensing.
3. A baseline scheme with fixed spectrum assignment was defined for comparison of the DSS techniques in terms of performance according to the following key performance indicators (KPIs): spectrum utilization, aggregated system throughput, packet error rate (PER), latency, system uptime, control overhead, etc.
4. The first demonstration of distributed DSS techniques for efficient spectrum sharing across multiple satellite systems (with ESIMs) and terrestrial networks, while protecting GSO and NGSO downlink and uplink transmission as well as terrestrial links.

The remainder of this paper is structured as follows: Section 2 presents an overview of state-of-the-art related to DSS and interference mitigation in SATCOM scenarios, Section 3 includes detailed information on the two selected use cases and problem statement, in Section 4 the proposed approach and requirements are defined, Section 5 comprises brief information on the proposed DSS techniques, Section 6 presents a simulation scenario and performance analysis of the DSS techniques against the baseline, and finally, Section 7 concludes the study.

## 2. Related Work

First, we are going to present the main spectrum sharing techniques used today in terrestrial networks. Similarly, as in SATCOM, sharing of the spectrum between terrestrial networks in licensed bands is characterized by static and fixed spectrum assignment. Licensed bands are being assigned for exclusive use by specific wireless operators or technologies. This approach leads to severe overprovisioning and underutilization both in time and geographically. In an attempt to alleviate this issue in licensed bands, licensed shared access (LSA) is introduced. LSA is a regulatory approach to allow any primary user to share its licensed spectrum with secondary users following a predefined sharing framework [11]. However, this approach is quite restrictive and will limit the quality of service that can be provided by secondary users. Furthermore, LSA access is applicable most of the time for a known set of two technologies that will coexist, hence it is not a generic solution that enables the transparent coexistence of multiple technologies. In unlicensed bands, for multiple technologies to share the same spectrum, listen before talk (LBT) schemes are used. LBT schemes are not scalable, and additionally, with large distances and propagation delays in SATCOM, they do not represent a suitable solution. Citizens broadband radio service (CBRS) [12] is a shared wireless spectrum from 3.5 GHz to 3.7 GHz. Devices that want to use the CBRS band requests from a spectrum access system (SAS) database to reserve unused channels in a particular geographic area. In the TV whitespace (TVWS) approach [13] in the 470–698 MHz spectrum range, secondary users are allowed to opportunistically access the spectrum unused by the primary TV services. In TVWS, access to the shared bands is managed by the centralized SAS database. Downsides of solutions based on databases are explained in detail later in the paper.

The recent work on coexistence in SATCOM scenarios can be categorized into two main groups: satellite-terrestrial coexistence and dual satellite coexistence. In satellite-terrestrial coexistence, the spectrum is shared between a satellite network and a terrestrial network, whereas in dual satellite coexistence two satellite networks operate simultaneously in the same spectrum band. Literature analysis shows that major research efforts on cognitive SATCOM sharing and interference mitigation are based on the construction and maintenance of centralized databases, where activity rules and main characteristics of primary users are stored. The key idea of a centralized database is that, before assigning a channel to a secondary user, the vacancy of the channel must be verified from the database for the duration of the targeted transmission period [14]. In the remainder of this section, we first present studies that investigated coexistence scenarios in satellite-terrestrial systems. Next, contributions in dual satellite coexistence scenarios are addressed. Finally, we present a couple of coexistence studies in both satellite-terrestrial and dual satellite systems that are based on database technique.

In [15], the authors proposed a joint power and subchannel allocation algorithm for the uplink scenario of the cognitive satellite-terrestrial network, where the cognitive GSO reuses the frequency band of incumbent terrestrial cellular networks in the S-band on 2.5–2.6 GHz. In [16] cooperative and cognitive radio (CR) techniques are applied to the satellite-terrestrial network operating in downlink and uplink in various frequency bands. The satellite segment consists of three GSOs, while the terrestrial segment is a 3G/4G heterogeneous network. Coexistence is achieved by cooperative spectrum sensing, where each earth station (ES) detects primary users’ transmitted signal and reports it to the satellite. Then, the satellite processes the received information from all the cooperative ESs and makes the final decision on the state of the spectrum, which is further broadcasted back to all the ESs. The focus of research in [17] is uplink communication in Ka-band where FSS satellite terminals try to reuse frequency bands of FS terrestrial microwave (MW) links which represent the incumbent users. Three different power allocation algorithms are used to control the transmit power of the FSS satellite terminals, thus keeping the aggregated interference caused at the FS system below some acceptable threshold. We can see from the presented papers on satellite-terrestrial coexistence that various cognitive techniques are applied in different frequency bands. However, all techniques focus on the static use case where the satellite segment consists only of GSO satellites and FSS to static ESs, and they cannot be applied to dynamic systems that, in addition to GSO satellites and terrestrial networks, may consist of NGSO satellites and mobile ESIMs.

Reference [18] proposes an adaptive power control technique for the coexistence of an NGSO satellite link with another NGSO/GSO satellite link for both downlink and uplink scenarios. The proposed technique is used to provide the required signal-to-noise ratio (SINR) at the receiver and mitigate the in-line interference between the GSO and NGSO systems. In [19], the cognitive power control from [18] was enhanced based on the distance between the NGSO satellite and the NGSO earth terminal. The focus of this study is on the mitigation of downlink in-line interference from the NGSO satellite to the static GSO earth terminal in Ka-band and improving link quality for the NGSO system. In [20], the operation of the cognitive network with GSO and low earth orbit (LEO) broadband systems is studied in the downlink Ka-band, where LEO satellites are incumbent users, whereas GSO satellites are secondary users. To enhance spectral efficiency and protect the incumbent system, an optimization algorithm based on beam hopping and adaptive power control techniques is proposed. While the proposed solutions offer coexistence for dual satellite systems, none of them can be applied to simultaneously improve spectrum utilization, mitigate interference, and enable coexistence in the most complex scenario where GSO and NGSO satellite systems (with static or mobile ESs) and terrestrial networks operate in the same frequency bands.

Reference [21] analyzes the case where interference is caused to GSO systems by terrestrial cellular systems and NGSO systems, respectively, both in the downlink and uplink Ka-band. The protection area is calculated where no cognitive users (terrestrial system or NGSO system) are allowed to transmit, whereas, outside the protection area, the cognitive users are allowed to transmit concurrently with GSO systems. However, it is worth noting that this paper analyzes two separate scenarios. In the first one, GSO is protected against NGSO interference, while in the other, against terrestrial interference. Therefore, coexistence is again not analyzed for the complex scenario where GSO, NGSO, and terrestrial systems operate together in the same frequency band.

Authors in [22] address the cognitive GSO uplink in the Ka-band where satellite terminals reuse frequency bands of FS terrestrial MW links which are the incumbent users. For the interference impact of GSO satellite on FS links to stay within the regulatory interference limitations, a joint power and carrier allocation strategy was proposed, which requires the existence of a complete and reliable FS database. Further extension of study in [22] can be found in [23], where a joint power and carrier allocation technique was proposed for uplink coexistence of GSO satellite with terrestrial FS in Ka-band. This technique was enhanced with a bandwidth allocation scheme that allocates bandwidth according to the user rate demands. This paper also analyzed the downlink coexistence of GSO and FS links in the Ka-band, where a joint beam-forming and carrier allocation was introduced. Availability of the FS database is assumed, which consists of FS antenna location and pointing directions.

In the CoRaSat project [24], Ka-band spectrum sharing between either single GSO or single NGSO (with static or mobile earth stations) and terrestrial users is mainly based on database techniques together with supplementary cognitive techniques as spectrum sensing. A centralized database and interference modeling is used to obtain the cognitive zones. Inside cognitive zones, opportunistic operations are only allowed with the use of cognitive techniques to reduce the interference, while outside these zones, entities in the network can transmit without restrictions. Cognitive zones are also applied in [25] for dual satellite FSS systems in 17.3–17.7 GHz band. The cognitive zone around incumbent broadcasting satellite service feeder links is determined by employing the characteristics of the links, which are stored and obtained from databases. Similar to the CoRaSat project, the cognitive FSS terminals can freely utilize the same frequency band outside cognitive zones. In the FREESTONE project [26], the possibilities to use frequency sharing techniques in SATCOM are investigated, and several application scenarios and use cases are defined. One of the use cases is the coexistence of terrestrial FS and satellite FSS systems in 17.7–19.7 GHz Ka-band. A database design is proposed, requiring information from the national FS registry, and information about the secondary FSS terminals. Based on the database, it is calculated where and how the FSS users are allowed to operate in the band. Authors in [27] extend on scenarios evaluated in the FREESTONE project and analyze sharing between GSO and NGSO satellite in Ka-band. They describe how a spectrum database could be utilized in controlling and assisting coexistence in such a scenario. It is required that systems’ main operational characteristics are stored in a spectrum database, such as frequency allocations, orbital positions, antenna patterns, etc.

It is worth noting that the introduction of dynamic entities like NGSO satellites or mobile ESs makes the use of a database approach very challenging [28]. Therefore, while solutions based on centralized databases may be appropriate for scenarios with a limited number of NGSO satellites (as assumed in all previously reported work), they might not be scalable to support upcoming large NGSO constellations. The downsides of approaches based on the centralized database are further investigated in Section 4. Similar to other studies on satellite-terrestrial and dual satellite coexistence, studies on coexistence techniques relying on databases have not yet addressed the problem of GSO, NGSO, and terrestrial networks simultaneously operating in the same frequency band.

## 3. Use Case Description and Problem Statement

The focus of this paper is on the opportunistic transmission of data using satellite links operating at the Ka-band. The terrestrial users coexisting with the satellite systems are considered as the incumbent users, whereas GSO and NGSO satellites are secondary users, with NGSO satellites having a lower priority than GSO satellites. In this study, we do not consider changing the spectrum management policies for the incumbent, but rather developing policies for GSO and NGSO entities so that the interference towards the incumbent is minimized. Therefore, only the satellite systems have DSS capabilities, whereas the terrestrial 5G Fixed Wireless Access (FWA) and MW links employ fixed spectrum allocation. The two most relevant and challenging use cases were selected: (1) opportunistic sharing of GSO/NGSO satellite and terrestrial systems in the downlink Ka-band; and (2) opportunistic sharing of GSO/NGSO satellite and terrestrial systems in the uplink Ka-band. These two use cases were selected based on the following criteria: (1) possible gains based on an initial qualitative evaluation; (2) importance and expected impact of the use case for the SATCOM industry; (3) complexity—coexistence of GSO satellites, NGSO satellites, ESIMs and terrestrial systems operating in same bands (4) implementation complexity; (5) need for new hardware support on satellites; and (6) need for new infrastructure on ground stations (GSs) and ESIMs.

### 3.1. Opportunistic Sharing of GSO/NGSO Satellite and Terrestrial Systems in Downlink Ka-Band

**Figure 1:**
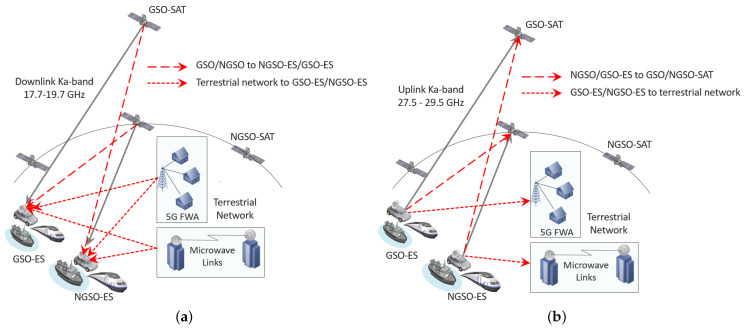
Selected use cases. (**a**) Downlink Ka-band. (**b**) Uplink Ka-band.

This use case concerns GSO and NGSO satellite systems and terrestrial systems in the downlink Ka-band (17.7–19.7 GHz) and is shown in Figure 1a. The terrestrial systems are mainly 5G FWA and MW links and it is assumed that they use frequency division multiple access (FDMA) for their transmission. The geostationary earth stations (GSO-ES) and non-geostationary earth stations (NGSO-ES) are mobile ESIMs, and they represent trains, ships, cars, etc. Furthermore, we consider that a control channel (CC) exists between different entities (satellites, ESIMs, GSs, and terrestrial networks). Since all the geostationary satellites (GSO-SAT), non-geostationary satellites (NGSO-SAT) and the terrestrial network operate in the same band, their transmissions can have an impact on the ESIMs. For the downlink operation of GSO and NGSO satellites in the presence of the terrestrial network, the following interference components are considered:

- GSO-SAT towards NGSO-ES;
- NGSO-SAT towards GSO-ES;
- Terrestrial network towards GSO-ES;
- Terrestrial network towards NGSO-ES;
- GSO-SAT towards terrestrial network;
- NGSO-SAT towards terrestrial network.

The goal here is to protect the communication of incumbent terrestrial networks, manage the interference towards GSO and NGSO ESIMs coming from GSO-SAT, NGSO-SAT, and the terrestrial network and improve the performance (spectrum utilization, throughput, PER, latency, etc.) by applying DSS techniques.

### 3.2. Opportunistic Sharing of GSO/NGSO Satellite and Terrestrial Systems in Uplink Ka-Band

This use case deals with GSO and NGSO satellite systems and terrestrial systems operating in the uplink Ka-band (27.5–29.5 GHz), as shown in Figure 1b. Similar to the downlink case, the terrestrial systems represent 5G FWA and MW links, and it is assumed that they use FDMA for their transmission. Furthermore, the assumption of a CC between different entities (satellites, ESIMs, GSs, and terrestrial networks) is applied here. Because of the spectrum sharing and possible spatial alignment of the ESIMs, it becomes possible that GSO, NGSO, and the terrestrial network are impacted by the transmission of the ESIMs. For the uplink case, we consider the following interference components:

- NGSO-ES towards GSO-SAT;
- GSO-ES towards NGSO-SAT;
- GSO-ES towards terrestrial network;
- NGSO-ES towards terrestrial network.

The goal here is to protect incumbent terrestrial networks, manage the interference towards GSO and NGSO satellites and the terrestrial network coming from the ESIMs, and improve the performance (spectrum utilization, throughput, PER, latency, etc.) by applying DSS techniques.

## 4. Proposed Approach and Requirements

In this section, we first motivate why we designed a distributed approach for applying DSS techniques and how this compares with more adopted centralized approaches. Furthermore, we define the main requirements and constraints for applying distributed DSS techniques on existing satellite/terrestrial systems.

### 4.1. Distributed versus Centralized Approach

In our study, we have adopted a distributed approach for dynamic spectrum access, as opposed to current more widely accepted centralized database approaches. The motivation to adopt a distributed approach rather than a centralized approach in this study is driven by the following observations:

- Driver: the main driver for a centralized database approach is to protect incumbents (primary users) while allowing opportunistic use of the incumbent’s spectrum by secondary users as long as the incumbent does not experience any harmful interference. The main drivers for a distributed receiver-based approach are scalability, by only reacting upon interference events detected at the receiver, and spectrum efficiency, by exploiting spatial reuse of spectrum.
- Mechanism: a centralized approach grants access to opportunistic users in a centralized way by consulting a global database that relies on transmission models of transmitters and the definition of exclusion zones (geographical areas within which active opportunistic radio transmitters are not allowed), restriction zones (geographical areas within which active opportunistic radio transmitters are allowed under certain restrictive conditions, limited by equivalent isotropic radiation power [EIRP] or EPFD), and protection zones (geographical areas within which incumbent receivers will not be subject to harmful interference). The distributed approaches that we adopted only take an action when interference is detected at the receiver. As interference only happens at the receiver of a communication link, the receiver is the best place to detect any possible interference, in other words, a receiver can measure the ‘ground truth’. A global centralized database, which is built based on transmitter models, cannot give more accurate information on the impact of interference than the local view as measured at the receiver. A model is by definition an approximation of the real world and always falls short of the complexities of reality. It is, for instance, very hard to accurately model multipath signal propagation, out-of-band emission, antenna properties (gain, side-lobe, back-lobe emission), Doppler shift, obstacles, weather conditions, etc.
- Spectrum monitoring: both approaches can be augmented with spectrum monitoring information. In the case of centralized approaches, geographically distributed spectrum monitoring devices are employed. A big issue here is accurate radio frequency (RF) calibration and abstracting hardware-specific features and artifacts of the spectrum monitoring device. Ideally, these devices should be capable of measuring the spectrum occupation for different directions around the spectrum monitoring device, increasing the complexity of the device. For comparison, in distributed approaches, when the spectrum is monitored at the receiver of a communication link using the same antenna configuration of the receiver, it will only capture relevant interference signals. Furthermore, there is no need for accurate RF calibration, as threshold levels for harmful interference can be perfectly calibrated through correlation with link statistics.
- Rules for spectrum sharing: to compensate for inaccuracies and deficiencies of the models applied in a centralized database, large safety margins are applied, leading to very conservative rules for spectrum sharing and hence largely limiting the benefits thereof. In distributed approaches, resources are dynamically allocated, triggered by interference events, which are detected by monitoring link properties at the receiver. The receiver is not only the best place to detect any possible interference, but also the best place to make decisions on the spectrum reallocation of the interfered communication link.
- Reaction time: consulting a centralized database for making decisions on spectrum resource allocation puts serious limitations on the reaction time, compared to local decisions using a fast dedicated CC.
- Scalability: centralized database approaches are not scalable when the number of involved entities is scaling up, due to the huge information exchange overhead to keep the database up to date. The number of exchanged messages that are needed to keep centralized databases up to date will further explode when dynamic entities are involved, which is the case with NGSO-SATs, ESIMs, and mobile users communicating with terrestrial systems. An increase in the dynamicity of the system may lead to outdated information stored in centralized databases. On the other hand, in distributed approaches, information exchange overhead between entities of satellite/terrestrial networks does not increase with the increase of network density. In this approach, information sharing between entities is event-based, information is exchanged only when required, which reduces control overhead and does not lead to performance degradation when network density increases.

### 4.2. Requirements

Since this study is aiming to redefine the framework of future SATCOM in order to achieve the highest possible spectrum efficiency, we are encouraged to identify and propose which aspects of SATCOM could be enhanced to be able to support dynamic spectrum slicing and allocation. Therefore, it is not necessary that the proposed framework follows existing standards and regulations in SATCOM. Rather, if achieved results are proven to be beneficial, they can serve as a basis for adaptation of existing SATCOM standards and regulations. Based on these considerations, the following requirements are identified throughout this work:

- A basic multi-frequency time division multiple access (MF-TDMA) medium access control (MAC) protocol is considered to be available and running in all satellite-related entities. This is the most basic enabler of spectrum slicing in its two dimensions (frequency, time) that is adopted by the broader wireless community (also in 5G) for future dynamic and spectrum efficient resource allocation. Although current satellite systems use frequency division duplexing (FDD) and further time division multiplexing (TDM) in downlink and MF-TDMA in uplink as in 2nd Generation Interactive Digital Video Broadcasting Satellite System (DVB-RCS2) [29], such a continuous TDM stream will be a major source of interference and this approach is not sustainable with the anticipated growth of satellite density. Therefore, we consider MF-TDMA for both downlink and uplink and elaborate its benefits in terms of spectrum utilization and interference avoidance capabilities.
- For the sake of simplicity, FDMA is considered for both 5G FWA and MW links. Note that the intention here is not to simulate the full stack of the terrestrial part, but rather to consider it as the incumbent.
- There is time synchronization between all entities taking part in the MF-TDMA based DSS framework with an accuracy at least in the order of 1 up to 2 ms, requiring that the distance between two extremes of the satellite’s beam, projected on the ground, is within bounds that allow the level of synchronization assumed. If terrestrial networks want to actively share spectrum in the time domain through the MF-TDMA MAC, then they also need to adhere to this time synchronization requirement.
- Each transmitter is capable of actively shifting its transmission bursts in frequency and in time in order to make sure that the receiver receives the transmission within the intended frequency range and time slot.

## 5. DSS Techniques and Generalized Finite State Machine

In this section, we discuss the DSS techniques that have been implemented and evaluated in selected use cases. We first introduce a generalized finite state machine (FSM) that works as the core of the DSS techniques, followed by a brief description of the two DSS techniques: (1) DSS1—collaboration protocol (CP); and (2) DSS2—decentralized spectrum sensing.

When designing DSS techniques, we have not focused solely on the two selected use cases and satellite operation in Ka-band. The DSS techniques we designed are generic and can be applied to other scenarios and in different satellite bands, such as S, C, X, Ku, etc. More specifically, they can be applied in situations where: (1) interference management is an issue due to the coexistence of other satellites/operators in the same spectral band; (2) improving spectrum utilization is required; and (3) distributed interference management is required. As the proposed DSS techniques are generic and agnostic to physical layer (PHY) technology (spectrum band, modulation, and coding techniques) and topology/constellations, they can be employed for solving the foreseen interference management and spectrum utilization problems in various use cases. Employing proposed DSS techniques for satellites operating in different bands would not require any modification of the DSS techniques, and we envisage that it would not lead to any performance degradation.

**Figure 2:**
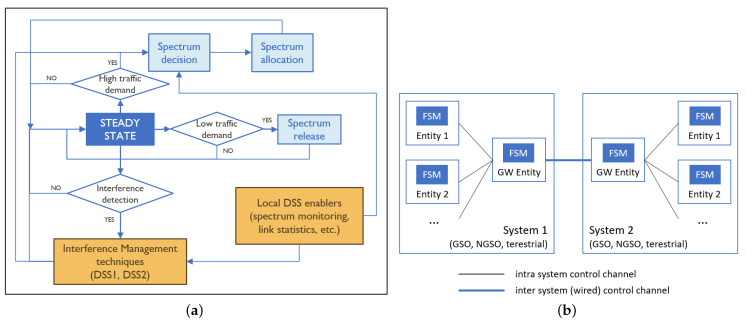
(**a**) DSS finite state machine (FSM) running on satellite/terrestrial entities. (**b**) CCs between system entities (GSO systems, NGSO systems, terrestrial networks) with FSM.

Figure 2 shows the generalized FSM and the generic entities, including gateway (GW) for the different systems considered in this study: GSO, NGSO, and terrestrial networks. Depending on the DSS technique, the operation of FSM at different entities can be mandatory or optional. Each entity that runs an FSM can collect local information such as spectrum occupation data (collected via spectrum monitoring), node position, antenna characteristics, etc., intra-system information from other entities operating within the same system, and inter-system information from entities operating in other systems. This information will be shared through established intra-system or inter-system CCs, depending on whether the entities communicate within or across systems. For instance, if the FSM operates on a GSO-ES, then it requires information from other GSO (satellite, ES), NGSO (satellite, ES), and terrestrial systems through their respective GSs/GWs and CCs.

The FSM includes the following building blocks: steady state, spectrum decision module, spectrum allocation module, spectrum release module, interference management techniques, and local DSS enablers. The starting point of the FSM is the steady state. While the system is in the steady state, as the dynamicity increases, the following events could happen: (1) low traffic demand; (2) high traffic demand; and (3) interference detection. In case of low traffic demand from the application side, the spectrum release module will be executed which will release some spectrum resources so that those resources become available for other entities, and the system will resume its operation in the steady state. In case of high traffic demand from the application side, the spectrum decision and spectrum allocation modules will be executed, which will allocate new spectrum resources for meeting the traffic demand. The job of the spectrum decision module is to find out the most suitable slots/channels to be used by GSO/NGSO satellites and ESIMs. It is the receiver of a communication link that proposes suitable slots/channels to the sender by utilizing: (1) local DSS techniques such as spectrum monitoring; and (2) transmission characteristics received from other entities that might interfere. The job of the spectrum allocation module is to implement different spectrum allocation and access mechanisms for: (1) protecting incumbent terrestrial systems; (2) satisfying the priority of GSO over NGSO systems; and (3) preventing interference among different GSO and NGSO transmissions. Lastly, the most important event from the steady state is interference detection, where interference can be detected in a variety of ways, such as SINR degradation, interference source detection, etc. All active links are monitored when the system is operating in the steady state, and in case of detecting an interference event, the interference management techniques, local DSS enablers, and spectrum decision module will be executed in order to move the system back to the steady state. Two DSS techniques have been designed for managing the interference and support flexible spectrum (re)allocation and release. These two DSS techniques are based on CP (called DSS1) and spectrum sensing (called DSS2).

### 5.1. DSS1—Collaboration Protocol

DSS1 is based on a decentralized CP that enables information dissemination between different node entities (within the same network or across different networks) that are taking part in the collaboration. This approach aims to provide each node with the necessary and relevant information about current spectrum usage and active transmissions from other nodes within its interference range. Having this information, a node can make informed decisions on its spectrum usage and avoid interfering with other nodes or getting interfered by others.

We further enhanced the CP protocol in DSS1 to support an optional alarm message. This alarm message is initiated by terrestrial nodes and advertises a problem when suffering from interference at the receiver. This allows satellite transceivers with link adaptive capabilities to protect terrestrial links that cannot adapt. Once the FWA link detects interference, it notifies all the ESIM and satellite transmitters participating in CP to reallocate slots that might be causing this interference. Upon reception of the alarm message, each entity in the network should filter it based on the active transmissions and react if necessary. The interfering satellite link will reallocate slots that directly overlap with slots allocated by the FWA link. In addition, slots that might cause adjacent channel interference (ACI) are also reallocated. The DSS1 technique based on CP enhanced with alarm messages is referred to as DSS1+ in the rest of the paper.

In DSS1 and DSS1+, the FSM shown in Figure 2a is executed at least in the GS (for satellite systems) or GW (for terrestrial systems), and optionally in the satellite and ESIM entities if decisions are taken locally at the satellite or ESIM. All the states explained in the generalized FSM remain the same with the DSS1/DSS1+ relying on the dissemination of necessary information via the CP. The main advantage of DSS1 is that it does not require any changes to the existing operation of satellite systems, and only requires the introduction of the CP that can run in the GSs controlling the satellites and ESIMs or the GWs controlling terrestrial links.

#### 5.1.1. Network Architecture of Collaboration Protocol

**Figure 3:**
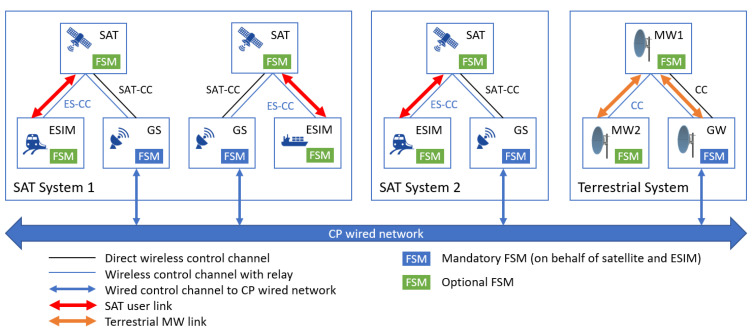
Network architecture of CP consisting of different entities with their respective user and control links.

The proposed CP network architecture, with multiple systems consisting of different entities and established user and control links, is depicted in Figure 3. As the focus of this work is not on feeder links, they are not presented here, however, all the approaches presented here can be extended to feeder links as well. The CP disseminates its CP messages between involved entities over a wired terrestrial backbone network via their GSs and GWs, supported by existing wireless CCs. The CP can be terminated in the GS and pass any decision to the satellite or ES via standard control messages over standard CCs: in the case of satellites, there is a direct satellite control channel (SAT-CC) between GS and satellite, whereas, in the case of ES, the earth station control channel (ES-CC) is relayed between GS and ES via the satellite. In this case, the GS will act as a CP proxy for the satellite and ES. Alternatively, by extending the standard CCs (SAT-CC and ES-CC) to pass special CP messages, the CP can also be executed on the satellite and ES, enabling them to take local decisions. The CP network is also connected to the terrestrial network, where GWs have a direct connection to the CP wired network, while other MW terminals will communicate via a terrestrial wireless CC (possible via relaying over MW mesh network).

#### 5.1.2. Collaboration Protocol Structure

CP is used to disseminate at least the following type of messages:

- Position of a node (mandatory message, generated by all transmitter nodes or their respective GSs/GWs);
- Active transmission characteristics (mandatory message, generated by all transmitter nodes or their respective GSs/GWs);
- FWA alarm message in case of detected interference (optional message, generated only by receivers in terrestrial networks or their respective GWs).

The position of a node can be defined in multiple ways, depending on the coordinate system employed. In our study, we used the Earth Centered Earth Fixed (ECEF) coordinate system [30]. Each node advertises its location periodically. To reduce the overhead of the GS-satellite control link, we assume that satellites rely on their GSs to advertise their position in the CP network.

Regarding the active transmission characteristics message, it is used to describe any active transmission from any interface of a transmitting node. Active transmission characteristics messages are advertised periodically upon change in transmission characteristics, if the change is substantial enough to influence other entities (i.e., additional spectrum allocated). Furthermore, the CP architecture is designed so that each active satellite and ESIM (or their GS) should have all relevant information only about transmissions that could affect them (in the case of FSM only running at GS, transmissions that could affect GS’s belonging satellites and ESIMs), their power levels, and their timing information, and therefore could build a synthetic interference map. For the transmission to be clearly described, and for receivers to be able to calculate the possible interference to them, the following information must be included in active transmission characteristics message for each interface:

- Transmission power setting of the interface;
- Destination focal center point coordinates;
- Gain as a function of transmission cone azimuth and elevation;
- Time information about the transmission (continuous, periodical, start and end of transmission, allocated MF-TDMA slot[s], etc.).

The FWA alarm message should contain the location of the receiver node and time-frequency resource usage information (i.e., utilized MF-TDMA slot[s]).

### 5.2. DSS2—Spectrum Sensing

DSS2 is based on decentralized spectrum sensing and it requires spectrum monitoring capabilities at each receiver taking part in the satellite network. There exist technical solutions to achieve continuous run-time wide spectrum sensing without the need for a time-sharing approach between normal operation and spectrum sensing. Spectra can be monitored at the receiver of a communication link using the same antenna configuration of the receiver, capturing only relevant spectrum usage information in the receiver’s immediate vicinity (spectrum usage that may affect directional satellite communication). By processing the raw I/Q samples, local energy maps can be created that can be employed for decision-making on spectrum allocation. In DSS2, the FSM from Figure 2a that includes spectrum sensing will be executed at the receiving entities.

DSS2 does not require a CP or any exchange of state/status data between different communicating entities, it only requires a dedicated CC between the communicating entities for slot (re)allocation or release. Upon detection of an interference event, each receiving entity, based on locally acquired spectrum monitoring information (local energy map), decides which free slots are available for mitigating interference. Then, over the dedicated CC, the receiver will alert the transmitter and initiate a slot reallocation procedure to replace the interfered slot with the new slots. Whereas DSS1 can only detect known interference from CP advertisements, DSS2 can detect any interference source at the receiver. However, DSS2 requires changes to the radio modem to integrate spectrum monitoring at the receiver. If existing radio modems on already deployed satellites cannot be upgraded, the DSS2 technique can only be implemented in new satellite systems.

### 5.3. Link Setup Procedure in DSS Techniques

Here we describe how a satellite-GS link or satellite-ES link will be established using the information provided from the CP and the synthetic interference maps (in DSS1) or local energy maps (in DSS2). As an example, let us assume that the link will be an uplink connection from GS or ES to the satellite. At the time that the link needs to be established, the GS/ES transmitter will alert through SAT-CC or ES-CC that it wants to set up a link with *X* slots (*X* will be determined from the incoming traffic volume) towards the destination satellite. Then, the satellite will examine its synthetic interference map or local energy map and, based on the interference levels depicted, will choose *X* interference-free slots and send them back to the GS/ES through the SAT-CC or ES-CC.

For DSS1, in case the CP information is not forwarded to the satellite or ES but is kept on the GS (serving the satellite or ES), the GS will decide on behalf of the satellite or ES based on the available information in the synthetic interference map and select the interference-free slots, informing the satellite or ES through the SAT-CC or ES-CC to switch its receiver into the selected slots. In any case, for DSS1, the synthetic interference map will be the main tool for picking up new MF-TDMA slots that are interference-free and can be allocated to the new link. In DSS2, by having a clear view of the channel occupation on the receiver side, all present sources of known or unknown interference can be avoided and therefore links can be set up with optimal link characteristics.

By defining a way in which transmitter and receiver of a new or pre-existing link can negotiate through a control link and reach a final agreement on slot allocation (employing their synthetic and local energy maps), the proposed DSS techniques can offer a completely decentralized solution of dynamic spectrum allocation and minimize interference.

### 5.4. Interference Detection and Avoidance Procedure in DSS Techniques

The procedure of interference detection and initiation of an interference avoidance procedure is presented in this section. For interference detection in both DSS techniques, we used PHY metrics like SINR, and MAC metrics like packet success rate (PSR), falling below a predefined threshold as an interference event trigger.

Let us analyze an example where a satellite-GS link or satellite-ES link is already established and starts to experience interference. At the time an interference event is triggered, the nodes participating in the link must take some actions to correct the link quality. Since it is the receiver that will identify the interference event, it is up to the receiver to consult its synthetic interference map (in DSS1) or local energy map (in DSS2) and pick new free and non-interfered slots to replace the ones that are getting interfered. The receiver will then alert the transmitter node to alter the schedule of the transmission by removing the interfered slots and enabling the new MF-TDMA slots. In case the GS is responsible for deciding on spectrum allocation, the satellite will just inform the GS about the interference event detection, and then the GS will decide to move the interfered slots to other time-frequency slots. Next, the GS will inform the satellite about the newly chosen slots. In both cases, the link will recover by abandoning the interfered slots and replacing them with new interference-free slots.

Let us also note that in case there is no MF-TDMA in place, but just an FDMA approach utilized on the link, the same link setup and interference avoidance procedures can be applied for channel selection instead of slot selection.

## 6. Simulation Setup and Results

First, the simulation scenario, baseline reference scenario, adopted assumptions and selected KPIs are presented. Afterward, the DSS techniques are compared to a baseline reference scenario with fixed spectrum allocation by running an extended set of simulations. Simulations were performed in a satellite environment based on the components and functionalities being reused and extended from Satellite Network Simulator 3 (SNS3) [31]. SNS3 is a satellite network extension based on the well-known ns-3 simulator environment [32].

### 6.1. Simulation Scenario

**Figure 4:**
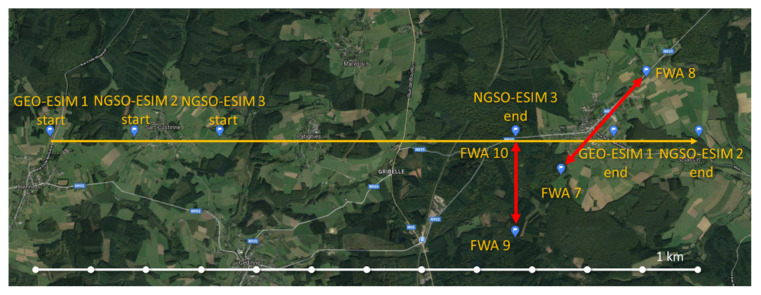
Simulation scenario on Earth’s surface.

Figure 4 shows the simulation scenario on Earth’s surface. It is a small-scale simulation scenario that includes three ESIMs (one GSO-ESIM and two NGSO-ESIMs) communicating to three satellites. ESIMs are moving in a straight line with variable speeds of 25–55 m/s (i.e., between 90 and 200 km/h, representing a range of typical train speed) between the ‘START’ and the ‘END’ points. We have assumed a straight-line motion of ESIMs in favor of implementation simplicity. However, ESIMs could follow any path without implication in the operation of the proposed techniques. With any arbitrary trajectory, achieved results would remain the same, as long as some interference events are triggered by ESIMs passing near the FWA links or being within the coverage area of each other when their beams spatially align. The reason for having three ESIMs in the collision domain (overlapping of satellite footprints) of the GSO-SAT and the two NGSO-SATs, and later on in the collision domain of the FWA links when they come closer to the FWA nodes, is to demonstrate at minimum the performance of the DSS techniques and how they manage to solve the interference when spatial separation is not possible. Further, there are two NGSO orbits at an altitude of 2000 km, an inclination of 50° from the Equator, and ascending nodes at 27.38 W and 142.61 W. In each orbit, only one NGSO-SAT is considered, as the scenario lasts 250 s and there is no need for link roaming between satellites. Both NGSO-SATs point towards their respective focal points around 50 N, 5 E, covering an area of 70–75 km around their focal points on the Earth’s surface.There is one GSO-SAT at an altitude of 36,000 km and it associates with one ESIM on the Earth’s surface. There are two point-to-point MW FWA links: (1) FWA 7–8; and (2) FWA 9–10. The first FWA link is parallel to the surface of the Earth, whereas for the second link, the elevation angle from FWA 9 to FWA 10 is 32° aiming to provide an alignment with the GSO-SAT.

The presented configuration of the simulation scenario is designed so as to trigger interference events between some of the participating entities per event. These are the generic interference events we aimed to create in this scenario:

- Alignment between ESIMs, NGSO-SATs, and GSO-SAT occurs around the 100th second of the simulation.
- ESIMs getting very close to FWA 10 and also aligning with the FWA 9–10 link.
- ESIMs passing through the FWA 7–8 link while also being very close to both transmitter FWA 7 and receiver FWA 8.

**Figure 5:**
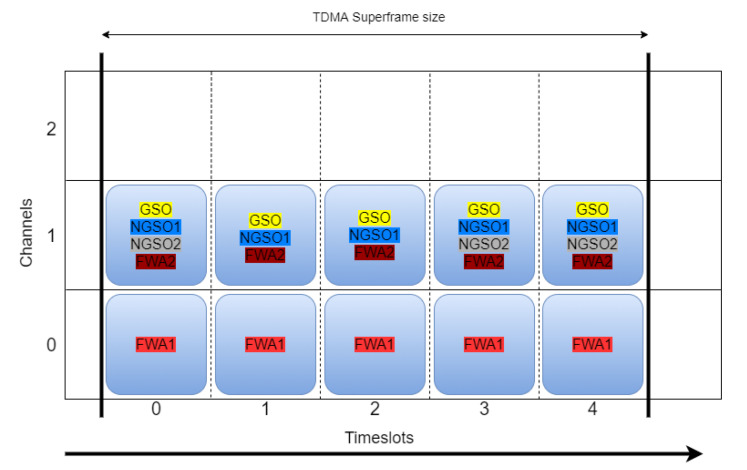
Initial spectrum allocation status.

All satellite links start the scenario by using channel 1. FWA 7–8 and 9–10 links use channels 0 and 1, respectively. By assigning the same channel to multiple links, the advantage of spatial separation of all nodes at the start is exploited, while enabling the detection of interference during the scenario in order to study the impact of the proposed DSS techniques. The initial spectrum allocation status is presented in Figure 5.

### 6.2. Baseline

With DSS techniques applied in satellite-terrestrial deployments, entities are able to move out of initially allocated slots. However, for benchmarking purposes, a baseline scenario without DSS is defined. For this reference scenario, all DSS mechanisms are disabled for the GSO and NGSO links, and static spectrum usage is assigned. Therefore, in the baseline, satellite-terrestrial systems keep initial spectrum allocation status throughout the simulation, in which all the involved entities, including GSO-SAT, NGSO-SAT, ESIMs, and terrestrial networks, operate in the same frequency bands. As motivated in Section 4, a basic MF-TDMA MAC protocol is considered for both downlink and uplink, running in all satellite-related entities. However, for the baseline scheme, restrictions are imposed on the MF-TDMA MAC scheme to comply with the current DVB-RCS2 satellite standard (TDM in downlink and MF-TDMA in uplink).

### 6.3. Assumptions

For the GSO and NGSO links, we consider up to four application flows with different throughput demands, which are enabled or disabled during the scenario. Therefore, the simulation scenario involves dynamically changing application loads for all satellite links. For the two FWA links, we consider a continuous application flow. The application layer packet size was selected to match the size of the PHY protocol data unit (PDU) (64,800 bits) to avoid fragmentation and aggregation requirements, as this was not the focus of this study. Specifically, the UDP/IP/MAC headers have a size of maximum 54 bytes, so the application layer packet was set to 64,368 bits. It must also be noted that each MF-TDMA slot supports sending a maximum of three PPDUs, and that the selected modulation and coding scheme (MCS) for all satellite communications was 16APSK, 2/3 coding rate, and 0.1 roll-off factor. The selected MCS requires an SINR of 9 dB in order to offer successful decoding of a PHY frame. Therefore, all satellite-terrestrial links are designed to have a reception threshold of 9 dB SINR, below which they lose connection. The nominal level of SINR is set to around 10 dB for satellite links, and around 20 dB for FWA links, offering a safety margin of 1 dB and 11 dB for satellite and FWA links, respectively.

Several other assumptions have been made to simplify the simulation scenario without heavily impacting the results:

- The simulations consider satellite antenna properties (main lobe, sidelobes, back-lobes, beam tracking, co-channel interference, and ACI) that are suggested in International Telecommunication Union (ITU) documents ITU-R S.672-4 [33], ITU-R S.1528 [34], and ITU-R S.580-6 [35]. ACI is modeled at −27 dB attenuated signal power compared to main channel power. Values for transmit power, pathloss, fading and other RF characteristics are adopted from SNS3 models.
- FWA RF characteristics are adopted according to [36].
- The simulator includes models for received signal strength indicator (RSSI) measurements and spectrum monitoring.
- For the reaction time of collaboration and resource allocation protocols, propagation delays are considered.
- Perfect modem synchronization, meaning zero time to reconnect after interference is solved.
- All satellite systems are considered single-beam systems.
- Feeder links are not simulated.
- A single carrier is adopted per PHY channel.
- ESIM antennas track the connected satellite.
- GSO/NGSO focal points are fixed.
- During the baseline scenario finetuning, a satisfactory PER threshold value for initiation of interference avoidance procedure is determined to be above 0.01%.
- The default MF-TDMA superframe consists of five time slots and 3–5 channels.
- The channel bandwidth of each channel is 12.5 MHz, consisting of actual 10 MHz signal usage and 2.5 MHz guard space. Guard spaces provide Doppler Effect protection, supporting speeds of ESIMs up to 2500 km/h.

### 6.4. Key Performance Indicators

In addition to SINR graphs, other KPIs measured through an extensive simulation study and used to evaluate the results are:

- Throughput is the aggregated application layer throughput achieved in each scenario run across all active links.
- PER (%) is the average application layer PER observed in each scenario run across all active links.
- Uptime (%) is the average percentage of simulation time that links were operational with 99.9% PSR and above across all active links.
- Spectrum utilization represents the percentage of allocated spectrum used for actual transmissions. Spectrum utilization of 100% means that all transceivers use their full allocated capacity (all allocated slots are used for transmission of 3 PPDUs).
- Latency is the average application layer latency observed in each scenario run across all active links.
- Wireless and wired protocol overhead of the DSS techniques in terms of information sharing via a control channel between GSO, NGSO, ESIM, and the terrestrial network.
  − CP overhead is the overhead per second induced from running the CP protocol across all wired and wireless links.
  − The slot selection protocol overhead is the overhead per second of the slot allocation protocol across all wireless links.

### 6.5. Results

All the KPIs are analyzed and presented for satellite and terrestrial links separately, in order to pinpoint the effects of DSS techniques on different networks in the system. In the end, three simulation sets were executed, two for uplink and one for a downlink scenario, each evaluating the two proposed DSS techniques and comparing them to baseline scenario.

### 6.6. Downlink Use Case

For the downlink use case, we analyzed the impact of NGSO/GSO downlink transmission and terrestrial links on the GSO and NGSO ESIMs. We also analyzed whether FWA links need protection during downlink transmission. Further, we examined the ability of the proposed DSS techniques to provide interference protection to the satellite links and, if needed, to terrestrial links. In this simulation set, the available spectrum consists of three channels.

#### 6.6.1. Baseline

**Figure 6:**
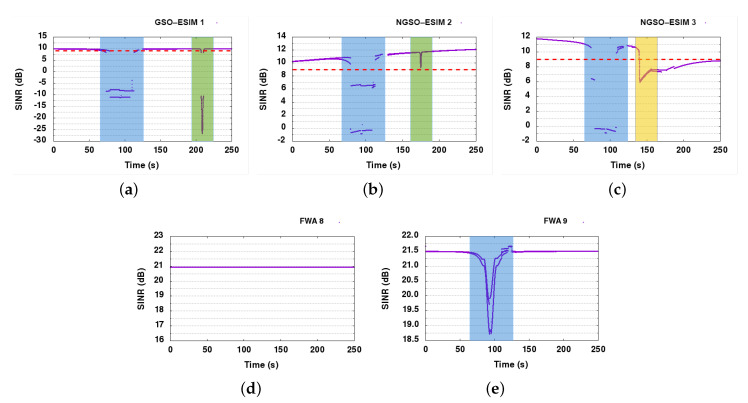
SINR graphs in the downlink use case (baseline). (**a**) GSO-SAT 1. (**b**) NGSO-SAT 2. (**c**) NGSO-SAT 3. (**d**) FWA 8. (**e**) FWA 9.

Figure 6 shows the SINR graphs of the FWA receivers, NGSO-ESIMs, and the GSO-ESIM. The FWA links experience an SINR drop of 2.75 dB in the case of FWA 9, and no drop for FWA 8. However, both FWA links remain operational, as the safety margin for correct reception SINR for them is around 9 dB. On the other hand, the GSO-ESIM receiver suffers on multiple occasions. In the presented SINR graphs, where measurements are dropping below the reception SINR threshold of 9 dB, this threshold is represented with a red dashed line. The first substantial dip below the reception SINR threshold of 9 dB (see dashed red line in Figure 6a is because of the alignment of all NGSO and GSO communication paths around the 100th second of the simulation run (see blue area). The second dip (green area) happens around the 210th second of the simulation time because of reaching the receiver FWA node 9. At that time, the GSO-ESIM receiver is perfectly aligned with the signal of FWA node 9, impacting its SINR and dropping the connection.

In SINR graphs of NGSO links, we can again distinguish the two major interference events, the NGSO-GSO geometry alignment at around the 80th second (blue area), and then the FWA 9–10 link interference effect at around the 170th second, as the ESIMs pass behind FWA receiver node 9. There is another slight impact for both of them around 170–200 s that corresponds to both of ESIMs crossing the second FWA link at different times. The impact of interference is substantially higher in NGSO-ESIM 3 (yellow area) than in NGSO-ESIM 2 (green area) because NGSO-ESIM 3 actually stops at the FWA receiver and thus suffers interference for a longer time than NGSO-ESIM 2.

The fact we can see two or more lines per SINR measurement is because of the condensed measurement of SINRs at the ms scale. We can see multiple levels of SINR measurements that are grouped per slot corresponding to multiple interference events impacting the signal. For instance, some slots might experience interference differently than others based on their ACI and co-channel interference. Hence, we often observe two or more lines in most SINR graphs.

**Figure 7:**
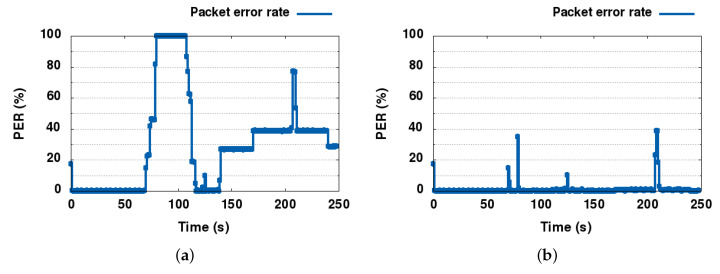
PER graphs for satellite links in the downlink use case. (**a**) Baseline. (**b**) DSS1.

In Figure 7a, the overall system PER graph is presented. It is clear that PER increases in the specific areas of the SINR graphs, where SINR drops below 9 dB for the GSO and NGSO links. NGSO 3 cannot recover after 120 s, as in the scenario it is designed that it stops close to FWA node 9 at that time and never moves again, therefore it is clearly interfered for the remaining of the scenario from the FWA link.

#### 6.6.2. DSS1

**Figure 8:**
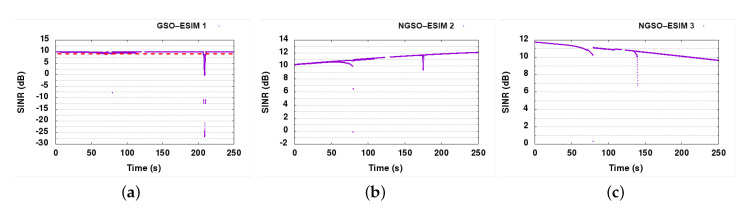
SINR graphs in the downlink use case (DSS1). (**a**) GSO-SAT 1. (**b**) NGSO-SAT 2. (**c**) NGSO-SAT 3.

Observing the SINR graphs of DSS1 in Figure 8, we can conclude that DSS1 is capable of resolving almost all interference events in the satellite links, since SINR drops below 9 dB for short periods before reacting and moving to free spectrum slots. There are, as it seems, enough slots for the three satellite links to not only avoid direct but also possible ACI from neighboring nodes. It is also clear that the nodes react only when the SINR drops below the error threshold. FWA node 9 in DSS1 is only losing 2.2 dB SINR, dropping from 22 to 19.8 dB, whereas FWA node 8 is only showing a loss of 0.1 dB in the worst case. We observed approximately 18.7 dB SINR in the baseline, marginally gaining 1.1 dB in DSS1 compared to the baseline. Since FWAs were not impacted negatively, and no big differences were observed in the results achieved with DSS, we did not present SINR graphs for FWAs. It is worth mentioning that in scenarios where FWAs links are heavily interfered by satellite communication, their protection would be guaranteed by applying the DSS1+ technique.

The PER graph explains even better how successfully DSS1 was able to solve the interference problems between the satellite links as shown in Figure 7b. The PER only spiked at the start of each interference event, being resolved less than a few seconds later.

**Figure 9:**
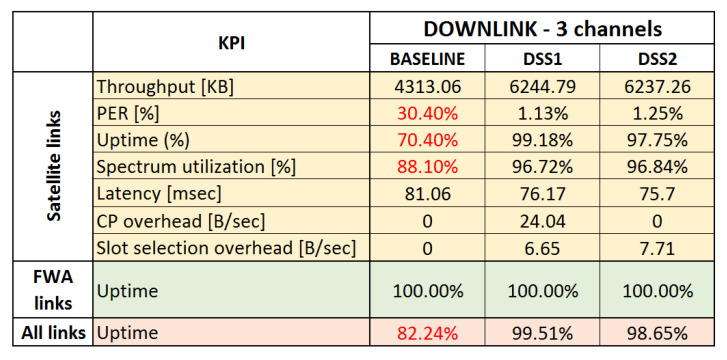
KPIs for the downlink use case.

Finally, the average overhead of the CP and slot allocation protocol can be seen in Figure 9. Both CP and slot allocation protocol overheads are minimal, only increasing when actions need to be taken to resolve interference events. After the bootstrapping period, the maximum CP overhead, both wired and wireless, is 500 bytes/s in total. That proves that no periodic exchange of information happens, except in the case of mobile ESIMs where they need to update their position information. The wireless slot allocation overhead is no more than 400 bytes/s in the worst case. The related CP advertisement messages and the slot allocation messages are all designed to be event-driven and therefore scale nicely with the network density.

#### 6.6.3. DSS2

Due to similarity with SINR graphs presented for DSS1, and to avoid duplication, we did not present SINR graphs for DSS2. Similar to DSS1, DSS2 manages to resolve all interference events on GSO and NGSO links, with SINR measurements keeping above the 9 dB threshold for the most part. All links are stable and are reacting when SINR drops below 9 dB within a few seconds. FWA nodes 8 and 9 do receive some interference from the downlink signals of the satellite links, but they stay within their safe margin, never dropping below 15 dB SINR. The FWA links had a 0% packet loss, like DSS1 and the baseline, as downlink communications do not seem to have a major impact on the Earth FWA links. If this impact is not negligible, DSS2 would not be able to protect FWA links, unless FWA nodes are supporting spectrum sensing and operating under DSS2 framework, which would make them adaptive and protected against external interference. It was found that DSS2 provides marginally lower packet loss and marginally better throughput in the dynamic part of our scenario, compared to DSS1. A closer look at our detailed logs reveals that DSS2 lost 1900 packets in total, while DSS1 lost 2000 packets, providing a small advantage, for a total loss in both of about 0.005% packets.

### 6.7. Uplink Use Case

In this section, we discuss the uplink use case and examine the impact of NGSO and GSO uplink transmission on the FWA links and the GSO and NGSO satellites. We also examine the ability of the proposed DSS techniques to provide interference protection to both the FWA and the satellite links during uplink transmission. For the uplink case, the FWA link direction between FWA 9 and 10 was reversed so that the FWA link now radiates towards the sky and not towards the earth. This allows us to analyze if the FWA links can be impacted by the GSO and NGSO ESIMs uplink transmission towards space.

In this simulation set, we allowed the system to allocate the spectrum into five available channels. An initial set of simulations was performed with three available channels, but it was noticed that DSS techniques cannot resolve interference because the two NGSO links are suffering from adjacent channel emissions from the GSO ESIM. It was observed that, although the NGSO links moved out of the channel/slots used by the GSO ESIM to avoid co-channel interference, the NGSO links still suffer a lot of interference in adjacent slots of GSO ESIM transmissions during alignment with the GSO. Such ACI can only be mitigated by moving away further from the GSO ESIM slots. For this reason, five channels are the minimum number of channels that allow NGSO links to avoid both ACI and co-channel interference from the GSO link.

#### 6.7.1. Baseline

**Figure 10:**
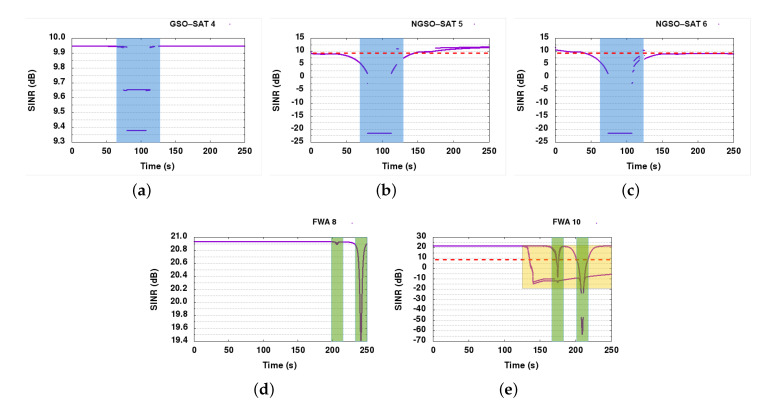
SINR graphs in the uplink use case (baseline). (**a**) GSO-SAT 4. (**b**) NGSO-SAT 5. (**c**) NGSO-SAT 6. (**d**) FWA 8. (**e**) FWA 10.

The SINR results for the baseline scenario simulation run are presented in Figure 10. As expected, the satellite connections are not stable in the baseline, as all links reuse the same channel for operation. During the convergence phase (see blue regions), both NGSO links suffer from interference but then recover to their normal operation. The GSO link is only slightly impacted by the interference from the NGSO links: we can observe that the SINR of the GSO receiver maximally decreases by 0.5 dB, which is within the 1 dB safety margin. The FWA links are impacted as the ESIMs are moving closer to them, aligning with the FWA beam. The impact on FWA 8 is much smaller, however, not more than 1.5 dB, whereas FWA 10 is seriously impacted by ESIMs passing close to its position. The interference impact of NGSO-ESIM 3 stopping behind the FWA 8 node is visible from 120 to 250 s of simulation time (yellow regions), whereas the passing of the other two ESIMs behind the FWA receiver is also quite visible in the SINR graph as deep spikes of SINR drops (green areas). Once interference occurs in the second part of the simulation run, the FWA links are heavily impacted and unable to operate.

#### 6.7.2. DSS1

**Figure 11:**
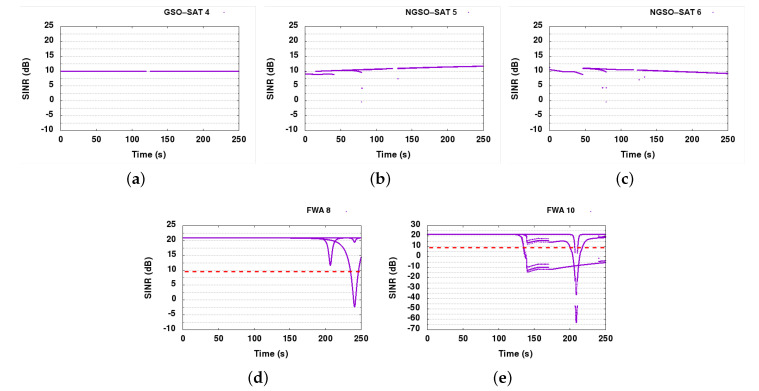
SINR graphs in the uplink use case (DSS1). (**a**) GSO-SAT 4. (**b**) NGSO-SAT 5. (**c**) NGSO-SAT 6. (**d**) FWA 8. (**e**) FWA 10.

DSS1 resolves a good portion of the interference events, especially in the satellite links. All satellite nodes (GSO-SAT 4, NGSO-SAT 5, and NGSO-SAT 6) are now healthy, maintaining SINR values over 9 dB in almost all cases throughout the simulation run (see Figure 11). FWA links continue to experience high interference, however, making it obvious that we were unable to protect them. Since the FWA nodes cannot dynamically select their channel usage, they cannot recover when interfered. The main problem comes from the GSO link that is very stable and does not suffer from any interference, and hence does not take any action for slot reallocation. To remedy this situation, we have envisioned DSS1+, an upgrade on the CP for DSS1 allowing FWA nodes to inform other systems participating in the collaboration about their interference status. With this upgrade, FWA links can alert other DSS capable nodes and trigger slot reallocation.

#### 6.7.3. DSS1+

**Figure 12:**
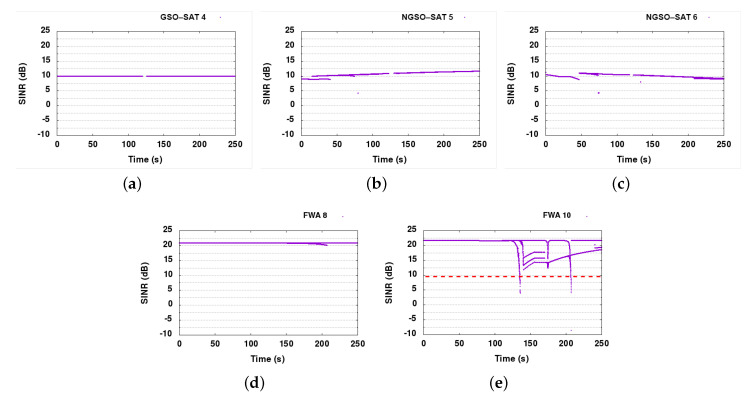
SINR graphs in the uplink use case (DSS1+). (**a**) GSO-SAT 4. (**b**) NGSO-SAT 5. (**c**) NGSO-SAT 6. (**d**) FWA 8. (**e**) FWA 10.

With the introduction of FWA alarm messages in CP of DSS1 technique, we can see from the SINR graphs in Figure 12, that FWA links are much better protected now. As there is no side effect of the extra FWA protection mechanism on satellite links, SINR graphs of satellite links are not presented here (same as in Figure 11). The protection mechanism offers a significant improvement, returning the FWA links almost back to optimal operation, with FWA links operating close to 99% uptime.

**Figure 13:**
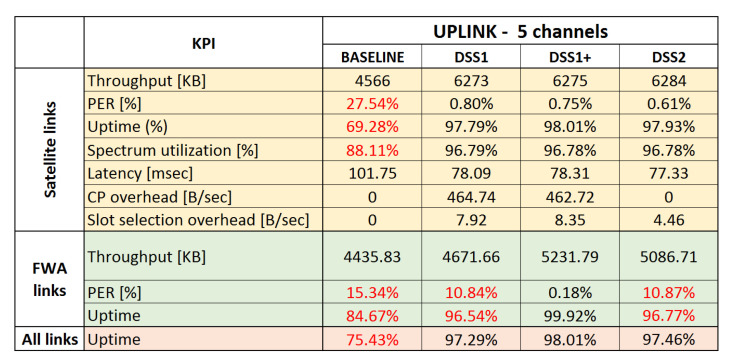
KPIs for the uplink use case, 5 channels.

The introduction of additional CP messages is also not causing a significant increase in control channel overhead. FWA alert messages are used sparsely, only in case interference is detected, in line with the event-based design approach of the CP. Hence, as expected, the induced overhead is minimal (see Figure 13).

#### 6.7.4. DSS2

As SINR measurement graphs are similar to DSS1, they are not presented here. Detailed analysis shows that with DSS2, the offered service was marginally better (see Figure 13). Satellite links behave almost optimally, however, just like DSS1, DSS2 fails to protect FWA links. When further examining the reason for this, we concluded that since DSS2 is based on spectrum sensing at the receiver, and the receiver of the GSO link is 36,000 km away, it cannot sense the presence of FWA links at the surface of the Earth. Furthermore, as the GSO link has zero PER, it does not need to initiate any slot reallocation procedure. On the other hand, the powerful GSO-ESIM transmitter creates a huge amount of interference on the Earth’s surface, impacting all links that are not capable of adapting their spectrum allocation. The only way to resolve the remaining issues using DSS2 is to also integrate the DSS module that supports spectrum sensing and dynamic spectrum access in FWA nodes, making them also adaptive and interference-aware. In this way, they would be able to simply move out of the spectrum that is affected by the GSO link.

### 6.8. Summary of Results and Main Findings

**Figure 14:**
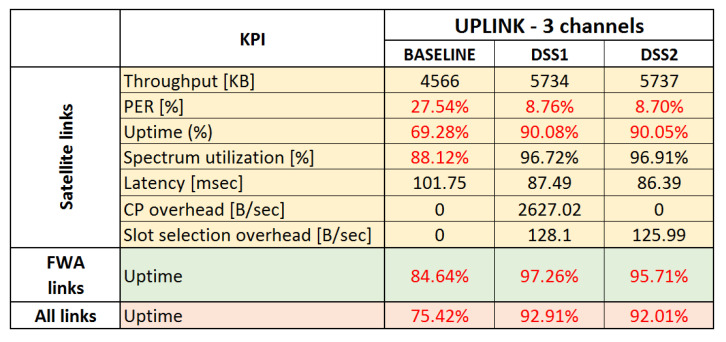
KPIs for the uplink use case, 3 channels.

A summary of the KPIs measured through this simulation study is presented in Figure 13 and Figure 14 for baseline and DSS techniques. For completeness, we also present results for the three channels uplink use case. Red numbers in the tables indicate unsatisfactory results. For the satellite links, the main focus was to benefit from opportunistic spectrum sharing with primary terrestrial links while not introducing harmful interference to the primary links, maximize uptime, and increase spectrum utilization by using collaborative and dynamic spectrum sharing techniques. As can be seen from the averaged results, avoiding interference may come at the cost of a short downtime (or reduced uptime) and a small packet loss during downtime.

The key results and findings for the two use cases are summarized below:

- Opportunistic sharing of satellite and terrestrial systems in the downlink Ka-band:
  − All DSS techniques offer significant advantages over the baseline in terms of link stability and uptime. All interference events can be detected by monitoring link statistics at the receiver. The overall uptime is increased from around 82.2% for the baseline scenario to 98.6–99.5% when DSS is applied.
  − All DSS techniques are able to quickly recover from interference generated by known satellite and terrestrial systems. When an interference event occurs, only a few packets are lost, and any event is resolved quickly by restoring a stable connection within a few seconds in the worst case.
  − Terrestrial MW FWA links do not suffer from interference by satellite links.
- Opportunistic sharing of satellite and terrestrial systems in uplink Ka-band:
  − All DSS techniques offer significant advantages over the baseline in terms of link stability and uptime. The overall uptime is increased from around 75.4% for the baseline to 98% when DSS is applied. 98% uptime can only be achieved when:
    * ACI is taken into account, in particular when high transmission power is involved, such as an uplink transmission by the GSO-ESIM. Additionally, side-lobe emissions can create interference events and should not be ignored.
    * Terrestrial MW FWA receivers send alert messages via the CP to warn satellite systems causing interference and to request them to move out of time-frequency slots also used by terrestrial FWA links.

Some additional key findings, independent of the use cases, are:

- Besides the capability of mitigating interference events, an additional advantage of using an MF-TDMA MAC scheme combined with proposed DSS techniques is the increased spectrum efficiency as the DSS techniques can instantaneously adjust the allocated spectrum to match the incoming application layer load, ensuring that no more than needed spectrum is used at any given time. It can be seen that in both downlink and uplink use cases, applying DSS techniques leads to an increase in spectrum utilization from 88% to 96%. We anticipate that the proposed DSS techniques would lead to much higher spectrum utilization gains in more complex and larger-scale satellite-terrestrial deployments.
- The wireless control overhead of all DSS techniques is negligible. The control overhead is only a few kB of data being exchanged per interference event between the related communicating entities, and remains constant per interference event, not scaling up with a number of deployed satellites, but with the limited number of satellites/ESIMs affecting or getting affected per interference event.
- In terms of implementation, DSS1 is the simplest technique, but it can only mitigate known interference sources (advertised by CP messages), whereas DSS2 with its spectrum monitoring capabilities can mitigate unknown as well as known interference.
- There is only one drawback with using event-driven DSS techniques: although the downtime is very limited, short periods with high packet loss of a few seconds are possible. For this simulation study, conservative link statistics measurements were used for detecting interference events. Interference was only detected after packets have been lost. This could be mitigated by applying techniques for the prediction of interference events (e.g., more advanced SINR analytics for early detection of SINR degradation or pattern recognition of recurring interference events using artificial intelligence and machine learning techniques) and proactive resource reallocation before packet loss occurs.

## 7. Conclusions

The current static and regulated spectrum allocation in SATCOM leads to underutilization of the spectrum. This regulated spectrum allocation cannot meet the emergence of numerous new satellite services and satellite operators that are bound to enter into SATCOM in the next few years. Therefore, to support a dense deployment of new satellite services and accommodate the new operators, it is required to dynamically share and reuse the finite available spectrum. The reuse and sharing of the spectrum can be enabled by employing DSS techniques. Two decentralized DSS techniques have been analyzed in terms of spectrum reuse for opportunistic sharing of satellite and terrestrial systems in downlink Ka-band and uplink Ka-band. The first technique relies on a decentralized CP that connects all entities across different systems and is used for advertising relevant information, while the second technique is based on decentralized spectrum sensing and requires spectrum monitoring capabilities.

Achieved results demonstrate that the proposed DSS techniques show remarkable interference mitigation capabilities as compared to the baseline, reducing the downtime of the established communication links by 60–99%. Interference mitigation between GSO and NGSO links is proven. Whereas FWA links are not affected in the downlink use case, protection of FWA links in the uplink use case was achieved with small upgrades to the DSS techniques. It has become evident that with a few more enhancements, a seamless experience can be provided to the application layer users in all cases. DSS techniques enable instantaneous adjustment of the allocated spectrum to match incoming application layer load, making sure no more than the needed spectrum is used at any given time. In conjunction with the use of a smaller basic transmission unit in the form of a slot instead of a channel, this enables increased spectrum efficiency, optimally mapping incoming traffic to the allocated spectrum. In both downlink and uplink scenarios, spectrum utilization was increased from 88% in baseline to 96% with applied DSS techniques. We anticipate seeing higher gains in spectrum utilization in the case of higher density satellite-terrestrial network deployments, where spatial reuse of spectrum plays a bigger role.

This work shows preliminary results of DSS techniques for enabling the coexistence of satellite and terrestrial networks. The evaluated scenarios are local and include a small number of satellite and terrestrial entities. In reality, there would be a higher number of satellite systems with their coverage areas including a much higher number of user terminals and terrestrial deployments. Therefore, in future work, large-scale testing is needed to investigate the scalability of proposed solutions and to pinpoint the areas where the design needs to be fine-tuned. Besides, with large-scale testing, we will be able to determine the impact of the proposed techniques in scenarios with large-scale satellite-terrestrial deployments. Another interesting point to discuss in the future is the improved protection of terrestrial links. Improved protection of terrestrial networks may be achieved, for instance, by improving CP alert message for anticipating packet loss or by extending the terrestrial networks with the same DSS techniques as applied in satellite systems. Besides, the anticipation of packet loss by applying advanced techniques for the prediction of interference events could enable proactive resource reallocation before packet loss occurs and further reduce downtime in satellite links. 

## Data Availability

Data available on request from the authors.
